# Food in Wartime

**Published:** 1917-10-13

**Authors:** 


### FOOD IN WARTIME.

### The Food Controller at Work.

It has been our good fortune to find in one of the famous West-Country institutions doing fine national service in this hour of stress a "food controller" whose methods are deserving of wide imitation. In the hands of a capable administrator housekeeping is seen to consist of countless details subordinated to two central principles—plenty of good food and no waste. And it is interesting to watch the interaction of these principles in the provision of abundant supplies at a minimum cost.

The "food controller" in question is honorary lady superintendent of an institution numbering over two hundred beds, who returned to duty, by request, from retirement when war broke out. No well-administered establishment waited till voluntary rations were instituted before reducing food expenditure, and here, as in many other places, a new and more strict economy has been practised since the first war winter.

### Purchase of Supplies.

Personal selection in buying provisions is considered of first importance in this establishment. The "food controller" finds it inexpedient to delegate this duty. However pressed by other duties she makes it her own business to sally forth each morning as soon as the shops open (8.30 to 9 a.m.) and cater for her household. Consultation with the cook the previous afternoon, and the lists prepared by those in charge of each section of the household, place her in possession of the quantities of perishable goods required to victual the household for the next twenty-four hours. She is accompanied always by one, sometimes by two probationers, who receive three months' kitchen instruction in cooking and general housekeeping duties as part of their training. The "food controller" has in her mind a general idea of the menu to be served on that and the following day, for both enter into her scheme. Seven distinct diet-tables for separate classes of inmates and staff have to be observed, so that it is no light task to calculate the required quantities. Such diets as are prescribed by medical order must, of course, be carried through practically without alteration. But the main menu for the day is subject to variation at a moment's notice, according to the state of the market. Should mutton prove dear on that particular morning, beef can be substituted. If the local fishermen have had a good catch, fish will be almost given away, and a fish dinner will be arranged. If stormy weather have sent up the price of fish, the usual fish dinner on one day in the week will be omitted, and so on. When contracts are entered into these variations cannot be made; the contractor, unless he be one in a thousand, will substitute meat of an inferior quality when the market goes against him, and the result is unsatisfactory all round. In times of scarcity the one important matter is to take the best thing available at the moment.

### A Cheap Dinner.

But there is another great advantage to be gained from personal shopping. The caterer deals with the principal, though not always at one shop or market-stall. Her custom is constant and her wants are large. Moreover she is dealing for a charitable institution, and if she be a person of discretion she will be able to enlist the sympathies of the salesman in favour of the charity. This will work out to the interest both of buyer and seller, provided always that the former be willing to study the convenience of the latter. No trades present greater difficulties as regards wastage from goods left on hand than that of the butchers, fishmongers, and greengrocers, from whom the main supplies of perishable goods are daily procured. The "food controller" can very easily provide a menu twice a week at an incredibly small price, even in these dear times, if she arrange to purchase *pieces* of beef, mutton, or veal from the butcher, as trimmed from joints to suit other customers or left unsold. The excellent cold-storage arrangements in all respectable butchers' premises render it possible to deliver these oddments of meat in sweet and sound condition. When stewed down the day before they are finally prepared for the table, they can be made into dozens of varied dishes which are all in higher favour from the hungry than perpetual roast and boiled. The particular favourite in the establishment to which we refer is Cornish pasty, well seasoned with onions, and few dishes are more savoury and nourishing. The price of "pieces" is not to be lightly divulged. Let any matron adventure forth in her own town and make inquiry for herself, sealing her lips to secrecy. She will not parley with her butcher in vain.

### Some Small Economies.

From the beginning of the war the edict went forth in this kitchen—no "swelter." The pig-tub remains empty, or rather the pig-tub itself has vanished. The good things which used to find a home in it are now consumed at table and voted excellent. There are the broad-bean pods, for instance. Everyone knows how small a dish of beans shells out of a big basketful as brought in from the garden. The "food controller" has borrowed a recipe from the canny Parisian and has them boiled, pods and all, till they are tender, merely cutting off the hard bit of stalk, and then fried in fat. The result is delicious, and the beans go more than twice as far as when the pigs got the pods. Pea-pods make excellent green-pea soup. Potatoes, when there is a scarcity, can be helped out by mashing them with an equal quantity of swedes or turnips, and the addition of a few onions renders the dish very palatable and popular. The potato-skins may be boiled till they are tender, sliced into thin strips and fried in fat. In this form they become potato chips and are much liked. As for fish-bones, they are first boiled down to make jelly and next buried. When thoroughly decomposed they make an excellent fertiliser for the garden. All sugar is liquefied before appearing at table, and may be said to go twice as far when thus treated.

### To Improve War Bread.

The reason why bread made from mixed flour is unwholesome is that the time of baking sufficient for the wheat particles does not thoroughly cook the maize constituents. The remedy is extra baking. The bread should be sprinkled over the top crust with milk, then steamed till quite moist, put into greased tins and set in the oven till it is baked right through and the crust is crisp. Consultation with the baker should remedy the defect at the source, but in no case should clammy, half-baked bread be issued to sick persons.

☛ Previous articles appeared February 24, March 10, 24, and 31, April 14 and 21, May 5, 12, and 19, June 9 and 16, and September 1 and 29.

